# Acupuncture therapy for persistent and intractable hiccups: Protocol of a systematic review and meta-analysis

**DOI:** 10.1097/MD.0000000000017561

**Published:** 2019-11-01

**Authors:** Han Yang, Rufei Zhang, Jun Zhou, Ying Cheng, Juan Li, Qiwei Xiao, Zihan Yin, Guixing Xu, Ling Zhao, Fanrong Liang

**Affiliations:** a College of Acupuncture and Moxibustion and Tuina, Chengdu University of Traditional Chinese Medicine,; b Affiliated Sichuan Provincial Rehabilitation Hospital of Chengdu University of TCM,; c College of Health Preservation and Rehabilitation, Chengdu University of Traditional Chinese Medicine, China.

**Keywords:** acupuncture, effectiveness, intractable hiccup, persistent hiccup, protocol, safety, systematic review

## Abstract

**Background:**

Persistent and intractable hiccups bring serious inconvenience to patients’ work and daily life, and impair their quality of life. Relevant studies showed that acupuncture therapy might be effective in treating persistent and intractable hiccups. However, there is no consistent conclusion so far. The aim of our research is to investigate the safeties and effectiveness of acupuncture in treating patients with persistent and intractable hiccups.

**Methods:**

We will search randomized controlled trials (RCTs) using acupuncture therapy to treat persistent and intractable hiccups in the following 6 English electronic databases and 3 Chinese electronic databases: MEDLINE, EMBASE, the Cochrane Library, Web of Science, PsycINFO, Allied and Alternative Medicine (AMED), Chinese National Knowledge Infrastructure (CNKI), Chinese Scientific Journals Database (VIP) and Wanfang data. The cure rate and the total effective rate will be considered as the primary outcomes. Complete cessation within a given period post-treatment of hiccups, changes in frequency or intensity of hiccups, concomitant symptom score, and adverse events will be considered as secondary outcomes. We will use Endnote software 9.1 for studies selection, Review Manager software 5.3, and STATA 13.0 software for analysis and synthesis.

**Results:**

we will synthesize current studies to evaluate the the safeties and effectiveness of acupuncture for persistent and intractable hiccups.

**Conclusion:**

Our study will provide evidence of acupuncture therapy for persistent and intractable hiccups.

## 1 Introduction

Hiccups (singultus), are known as an involuntary, spasmodic contraction of the diaphragm and intercostal muscles somewhile which causing air disturbance, followed by the abrupt closure of the glottis.^[1]^ According to the duration, hiccups are classified as acute (≤48 hours), persistent (48 hours-1 month), and intractable (>1 month).^[2]^ It was reported that in the US, roughly 4000 patients require hospitalization for managing hiccups per year.^[3]^ Sometimes hiccups are considered trivial because they are often self-limiting, while they can be persistent and intractable occasionally, which are torturous to some patients. Persistent and intractable hiccups can be a manifestation of deep-seated pathology,^[4]^ correlating with gastrointestinal diseases, central nervous system, primary pulmonary and cardiovascular problems, psychogenic problems, infection, and etc.^[5–7]^ It has an impact on the daily life of patients, such as insomnia, anxiety, exacerbation of malnutrition, aspiration, pneumonia, wound dehiscence (especially in postoperative patients), and even death in the extreme condition.^[1–3]^

Drugs, such as chlorpromazine, metoclopramide, baclofen, haloperidol, gabapentin, and benzodiazepines have been used in persistent and intractable hiccups. Owing to undesirable adverse reactions, doctors and patients are seeking for non-pharmacological therapy. Acupuncture is one of the non-drug related treatments for persistent and intractable hiccups, which is widely adopted in China. It has been reported that acupuncture therapy may work in persistent and intractable hiccups.^[8–11]^ In 2016, a systematic review and meta-analysis^[12]^ concluded that acupuncture was effective on intractable hiccup. However, due to methodological defects, the conclusions are not reliable. At present, there is no specific evidence that ensured the effectiveness and safeties of acupuncture on this topic. The aim of our research is to evaluate the effectiveness and safeties of acupuncture therapy for persistent and intractable hiccups.

## 2 Methods

### 2.1 Study registration

The protocol of our study has been registered on the International Prospective Register of Systematic Reviews (PROSPERO) (registration number, CRD42019142331). The protocol is conducted strictly based on the Preferred Reporting Items for Systematic Reviews and Meta-Analyses Protocols (PRISMA-P) guidelines.

### 2.2 Eligibility criteria

#### 2.2.1 Type of study

The randomized controlled trials (RCTs) of acupuncture therapy for persistent and intractable hiccups will be included.

#### 2.2.2 Type of participant

We will include the study involving participants diagnosed with persistent or intractable hiccups (duration lasting > 48 hours)^[13]^ with or without organic lesions. There is no restriction on age, gender, or race.

#### 2.2.3 Type of intervention

We will include studies in which intervention group using acupuncture therapy (acupuncture, auricular acupuncture, electro-acupuncture, scalp acupuncture, warm acupuncture, dry needling, acupoint injection, press needle, acupressure, acupoint catgut embedding, etc) as the main intervention, and the control group using non-acupuncture therapy (western medicine, traditional Chinese medicine, conventional treatment, placebo, or waiting-list) or sham acupuncture.

#### 2.2.4 Types of outcome measurements

Primary outcome

(1) The cure rate (number of people curative/total number of people treated);
(2) The total effective rate (number of people curative and effective/total number of people treated).

We define curative effect^[14]^ as following: curative: after treatment, hiccups cease, and other accompanying symptoms disappears; effective: shorter duration of hiccups or decreased frequency of hiccups or it just recurred occasionally after treatment; invalid: after treatment, there are no observable changes for duration and frequency of hiccups.

Secondary outcomes

(1) Complete cessation within a certain time frame post-treatment of hiccups;
(2) Change in frequency or intensity of hiccups;
(3) Concomitant symptom score (anxiety, insomnia, tension, respiratory symptoms, and anxiety; 0–4 points to indicate no, light, medium, serious, very serious).^[15]^
(4) Adverse events related to interventions.

#### 2.2.5 Exclusion criteria

The diagnosis of the subjects not clear will be excluded; studies with acupuncture therapy not selected as the main treatment in the intervention group will be excluded; the unavailable full text will be excluded; duplicated data will be excluded; data cannot be extracted will be excluded.

### 2.3 Search methods for identification of studies

#### 2.3.1 Electronic data sources

The following 6 English electronic databases and 3 Chinese electronic databases from inception to October 2019 will be searched: MEDLINE, EMBASE, the Cochrane Library, Web of Science, PsycINFO, Allied and Alternative Medicine (AMED), Chinese National Knowledge Infrastructure (CNKI), Chinese Scientific Journals Database (VIP) and Wanfang data.

#### 2.3.2 Other resources

Relevant references will be reviewed and screened. Moreover, the following registration website of the clinical trial will be searched: WHO ICTRP, http://www.chictr.org.cn, http://www.ClinicalTrial.gov, and ISRCTN Register. In addition, the relevant grey literature will be searched from the Health Management Information Database (HMIC), OpenSIGLE Database, and the National Technical Information Service (NTIS). Experts in the field will be consulted for relevant researches.

### 2.4 Search strategy

Following the principle of combining subject words with text words, we will search the electronic databases with search terms for acupuncture therapy, persistent and intractable hiccups, and randomized controlled trial. There will be no restriction on languages and publication dates. Taking MEDLINE as an example, the specific search strategy is as Table 1. We will modify the search strategy according to the characteristics of different databases.

**Table 1:**
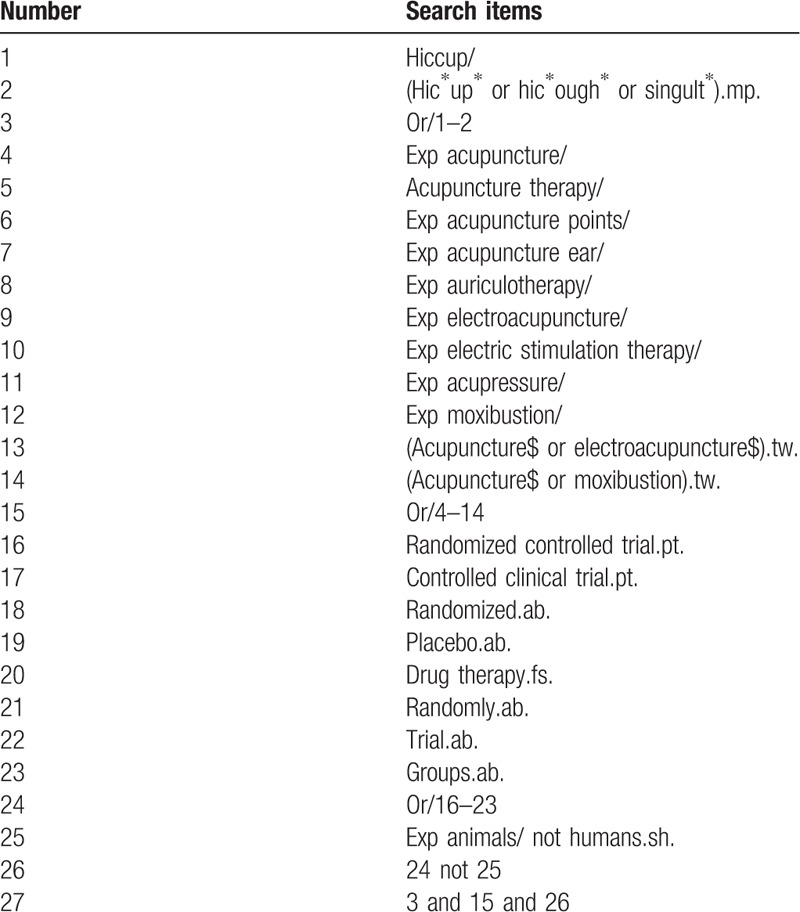
Search strategy for the MEDLINE database.

### 2.5 Data collection

#### 2.5.1 Selection of studies

**Figure 1:**
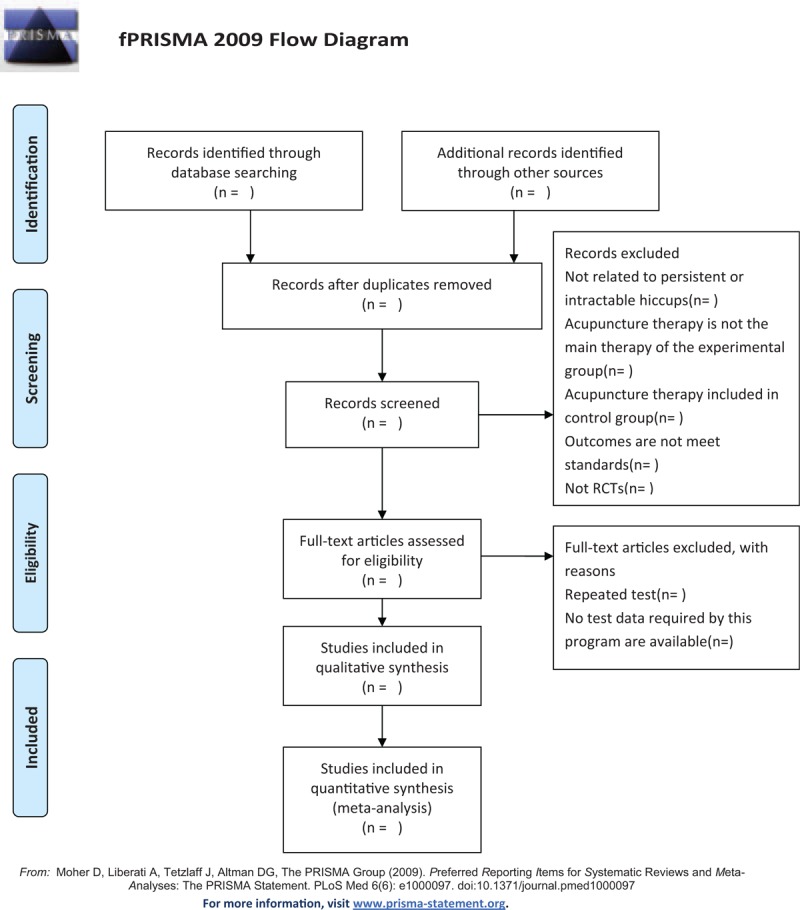
The Preferred Reporting Items for Systematic Reviews and Meta-analyses Protocols flow diagram of the study selection process.

The retrieved researches will be imported in Endnote software 9.1 to remove duplicates. The abstracts and titles of 2 independent researchers (JZ and QWX) will be screened according to the established inclusion and exclusion criteria, after that, we will screen the full text for the second selection. Two researchers (JZ and QWX) will cross-check the included studies. In case of disagreements, the third researcher (FRL) will be involved. The detailed screening process will be shown in the following PRISMA-P flow diagram (Fig. 1).

#### 2.5.2 Data extraction and management

The pre-designed form will be filled by 2 independent researchers (GXX and ZHY) and the extracted information includes: author, publication year, country, characteristics of participants, hiccups type, concomitant diseases, the details of intervention and comparisons, outcomes and the specific data, adverse events, follow-up, results, conclusions, sources of funds, conflicts of interest, ethical approval and methodological quality. Two researchers (GXX and ZHY) will cross-check the data extraction. In case of disagreements, the third researcher (FRL) will be involved. We will contact the author for more information if necessary.

#### 2.5.3 Assessment of risk of bias in included studies

The risk of bias of each included RCT will be evaluated by 2 independent researchers (HY and JL) according to the guidance from the Cochrane Handbook of Systematic Reviews of Interventions.^[16]^ We will evaluate the risk of bias from the following 6 domains: selection, performance, attrition, detection, reporting, and other sources of bias. The risks of bias will be marked as three levels: low (meet all criteria), high (meet none of the criteria), and unclear (trials with insufficient information to judge). Two researchers (HY and JL) will cross-check after assessment. In case of disagreements, the third researcher (FRL) will be involved.

### 2.6 Data synthesis

If there is a possibility to perform a meta-analysis, we will use Review Manager software (RevMan5.3) and STATA 13.0 software to conduct all data analyses. The random-effect model will be selected to analyze all the data. Descriptive analysis will be conducted if there is significant statistical heterogeneity.

#### 2.6.1 Measures of treatment effect

Mean difference (MD) will be used to evaluate if the extracted date for continuous outcomes (the complete cessation within a certain time frame post-treatment of hiccups, change in frequency or intensity of hiccups and concomitant symptom score) have the same unit. Otherwise, standardized mean difference (SMD) will be used. For dichotomous outcomes (cure rate, total effective rate, and adverse events), the number of participants, rate ratio (RR) will be used to analyze. For both continuous outcomes and dichotomous outcomes, we will set the confidence intervals (CIs) to 95%.

#### 2.6.2 Management of missing data

We will contact the corresponding author for detailed information for any missing or insufficient data. We will exclude these researches if we still cannot get accurate data after contacting the corresponding author.

#### 2.6.3 Assessment of heterogeneity

Heterogeneity will be assessed by qualitative analysis (comparing the included studies’ characteristics) and quantitative analysis (using the *I*^2^ test and the Chi^2^ test). When *I*^2^ < 50%, small heterogeneity will be prompted. When I^2^ ≥ 50%, significant heterogeneity will be prompted.

#### 2.6.4 Assessment of reporting biases

Funnel plots will be used to examine the potential publication bias if there are no less than 10 RCTs included. Otherwise, egger's test will be used to examine the potential publication bias by STATA 13.0 software.

#### 2.6.5 Subgroup analysis

We will perform subgroup analysis based on the different type of hiccups, hiccups with different etiologies, different kind of acupuncture therapy applied, different interventions of the control group and different time points for evaluating outcome after treatment.

#### 2.6.6 Sensitivity analysis

Sensitivity analysis will be performed according to the missing data, risk of bias, and sample size to evaluate the robustness if there is significant statistical heterogeneity.

### 2.7 Grading the quality of evidence

The quality of evidence of each outcome are ranked into 4 levels (high or moderate or low or very low) through assessing the quality by the Grading of Recommendations Assessment, Development, and Evaluation (GRADE)^[17]^ from the following 5 aspects: limitation of study design, inconsistency, indirectness, imprecision, and bias of publication.

### 2.8 Ethics and dissemination

Since our study have no connection with individual patient data, thus, it is not necessary for ethical approval. The results of our systematic review will be reported on a peer-reviewed journal or relevant conferences to provide the implication of acupuncture therapy for patients with persistent and intractable hiccups.

## 3 Discussion

Persistent and intractable hiccups bring serious inconvenience to patients’ work and daily life, damaging their quality of life. So far, there is no satisfactory treatment for persistent and intractable hiccups. Acupuncture treatment might be effective. Although 1 study^[12]^ concluded that acupuncture was effective on intractable hiccup through systematic review and meta-analysis, but its quality of the methodology was extremely low according to AMSTAR 2 scale^[18]^: there was no explicit statement on the review methods established prior, nor comprehensive literature search strategy and so on, affecting the credibility of the conclusion. So, the validity is still unclear. We will conduct this systematic review to evaluate the effectiveness and safeties of acupuncture therapy for persistent and intractable hiccups strictly following the Cochrane Handbook for Systematic Reviews of Interventions,^[16]^ to provide different perspectives and guidance for doctors.

## Author contributions

**Conceptualization**: Han Yang, Jun Zhou.

**Methodology**: Han Yang, Rufei Zhang, Juan Li, Jun Zhou, Guixing Xu, Zihan Yin.

**Supervision**: Fanrong Liang.

**Writing – original draft**: Han Yang, Ying Cheng, Qiwei Xiao.

**Writing – review & editing**: Han Yang, Rufei Zhang, Juan Li, Ling Zhao.
